# Osteoid Osteoma of the Trapezium: Case Report of an Unusual Tumor Location Presenting a Diagnostic Challenge

**DOI:** 10.1155/2017/3683854

**Published:** 2017-12-31

**Authors:** S. E. Roberts, M. N. Mirzabeigi, A. Naik, C. Preciado, B. Chang

**Affiliations:** ^1^Division of Plastic Surgery, Perelman School of Medicine, University of Pennsylvania, Philadelphia, PA, USA; ^2^Division of Pathology, Perelman School of Medicine, University of Pennsylvania, Philadelphia, PA, USA

## Abstract

Osteoid osteoma is a benign bone tumor, which represents approximately 10% of all benign bone tumors. When localized to the carpus, osteoid osteomas are most often seen in the scaphoid and capitate. Rarely, these tumors can also be observed in the trapezium. Given the infrequency with which osteomas are located in the trapezium and often nonspecific presenting symptoms, diagnosis of this tumor can be challenging and requires a high index of suspicion.

## 1. Introduction

Osteoid osteoma is a benign bone tumor, which represents approximately 10% of all benign bone tumors. Osteoid osteomas have been previously reported in the carpus [1–4] but rarely are these tumors found in the trapezium [5–8].

## 2. Case Report

A 34-year-old right-hand dominant female, office manager, presented with a two-year history of pain at the base of the right thumb and radial-sided wrist. She denied any trauma to the hand. The pain would sometimes wake her from sleep. She previously saw two surgeons and was told that her pain was likely due to arthritis. She had been taking naproxen regularly, which provided temporary pain relief.

Examination of her hand and wrist demonstrated hypersensitivity along the radial side of the wrist and along the right thumb with pain radiating up to the IP joint. The remainder of her exam was normal.

**Figure 1:**
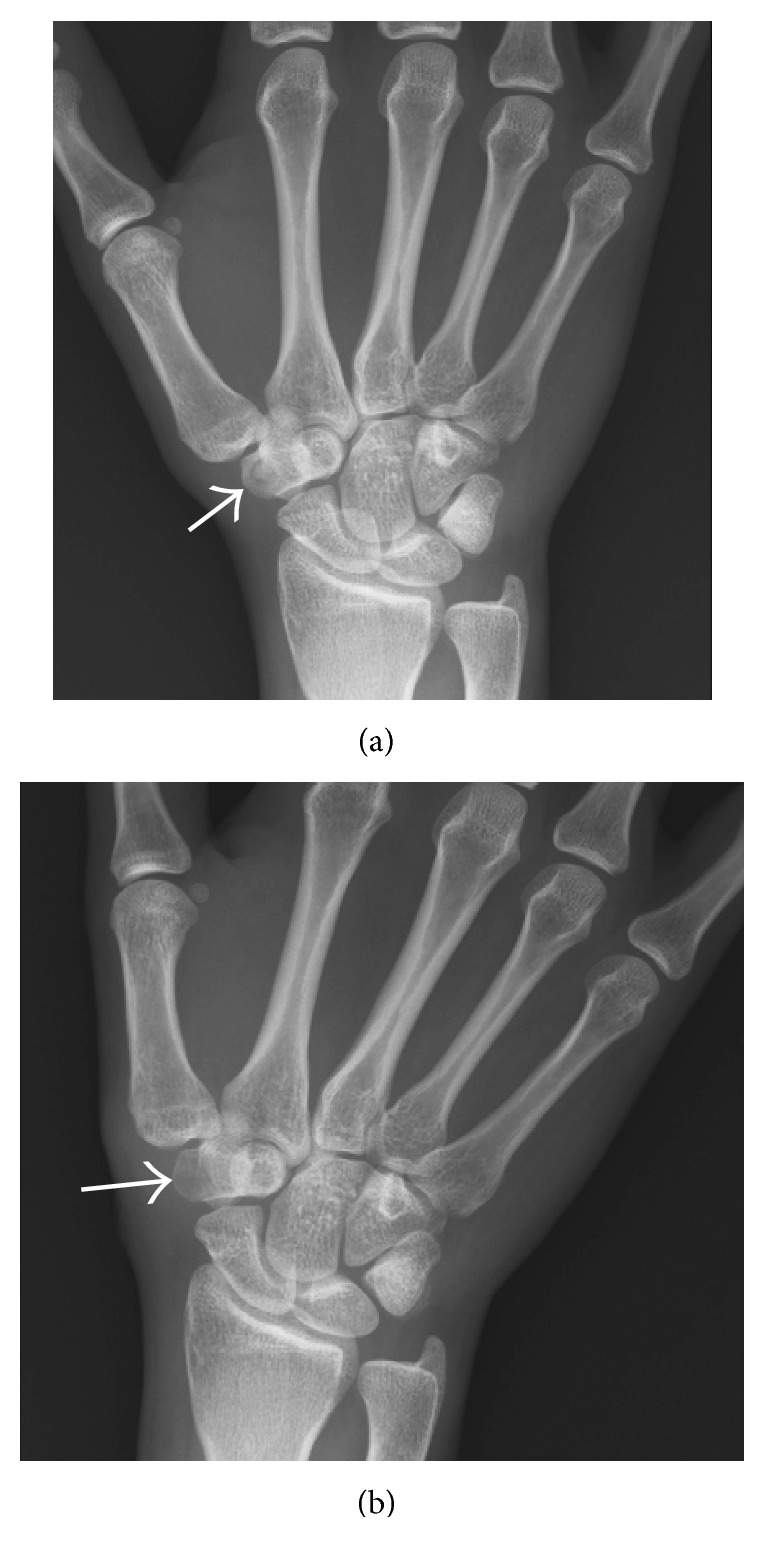
(a) Preoperative radiography of the wrist showing nidus (arrow). (b) Postoperative radiography of wrist.

**Figure 2:**
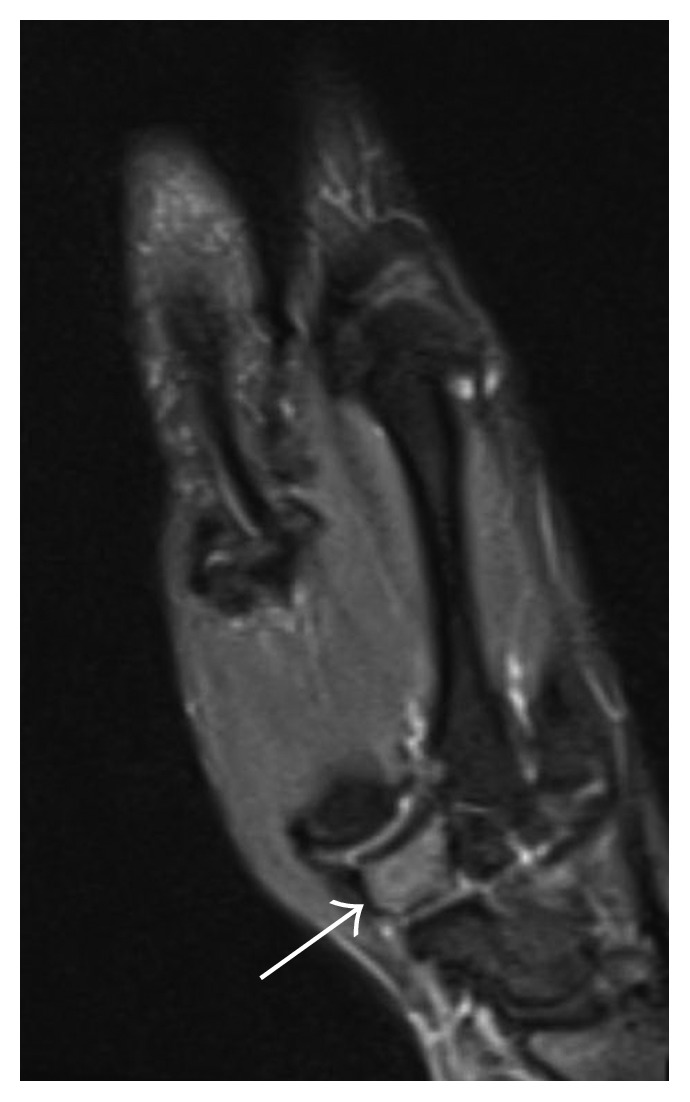
Magnetic resonance imaging demonstrating circular hypointense lesion (arrow).

**Figure 3:**
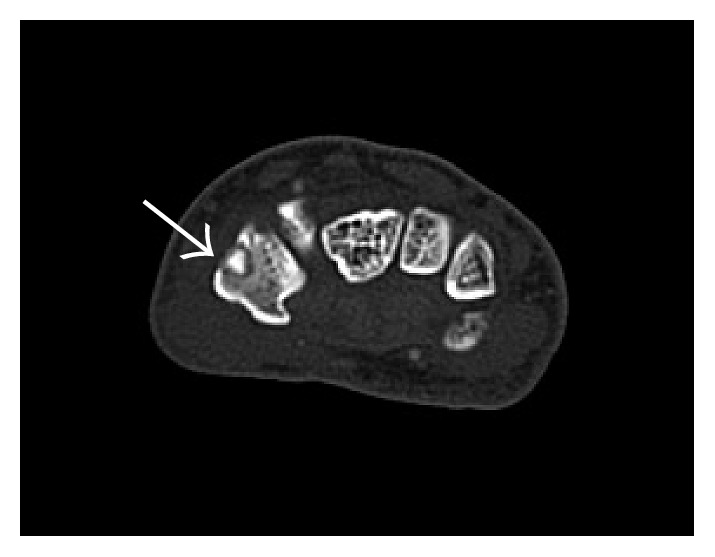
Computed tomography showing sclerotic nidus surrounded by radiolucent halo (arrow).

Initial radiographic films (Figure 1) were read as unremarkable; however, given high clinical suspicion of osteoid osteoma, an MRI was obtained which demonstrated a circular hypointense lesion along the dorsal aspect of the trapezium (Figure 2) suspicious for an osteoid osteoma. A CT was ordered to further characterize the lesion. CT imaging demonstrated the same defect yet slightly larger (Figure 3).

**Figure 4:**
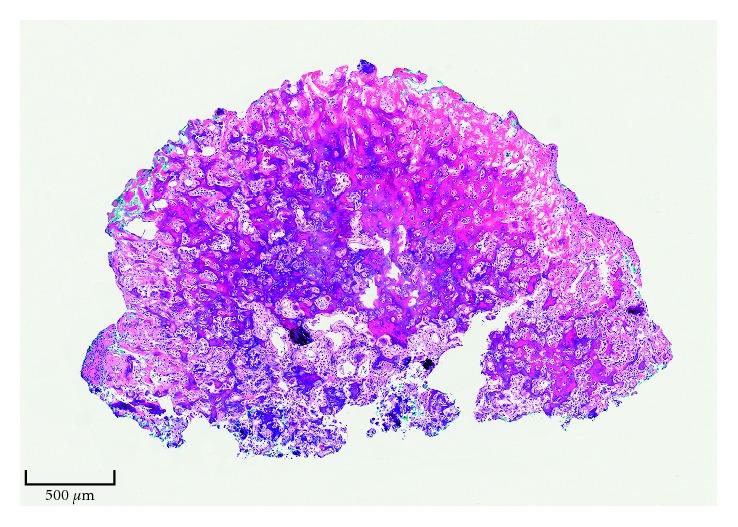
Photomicrograph of the osteoid osteoma. The tumor is composed of haphazardly interconnected trabeculae of osteoid and woven bone (hematoxylin and eosin; original magnification, ×5).

**Figure 5:**
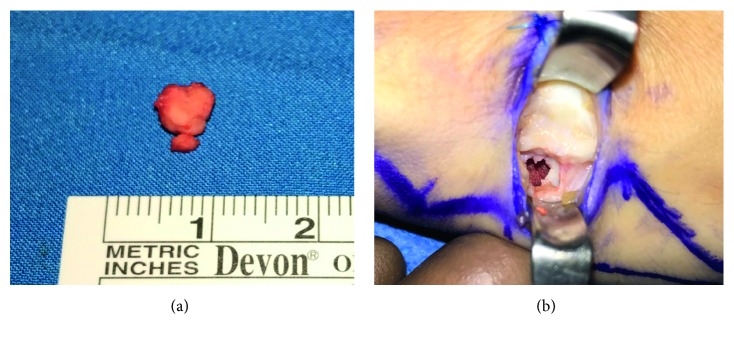
(a) Gross images of the explanted tumor. (b) Intraoperative photograph of the surgical site after tumor removal.

Surgical resection was planned through a 2 cm vertical dorsal incision. The lesion was marked intraoperatively with C-arm fluoroscopy, and an en bloc excision was done. Histopathology confirmed the diagnosis (Figure 4), and intraoperative fluoroscopy confirmed complete removal of the lesion (Figure 5). Given the central location of the lesion within the trapezium, small lesion size, and stability of the bone after en bloc removal, a bone graft was deemed unnecessary.

The patient has reported complete pain relief since her surgery. She had no recurrence to date.

## 3. Discussion

Osteoid osteoma presents with pain in 80% of patients; often, this pain is worse at night and relieved with salicylates or other nonsteroid anti-inflammatory agents [9]. The pain from osteoid osteomas arises from the production of prostaglandins from the lesion. While the exact pathophysiology is not fully understood, it is theorized that prostaglandins may directly stimulate free nerve endings inside or close to the tumor and lower the nociceptive threshold [10]. Salicylates or NSAIDs likely provide pain relief via inhibition of prostaglandin production.

Osteoid osteoma often presents during the second or third decade of life. Males are twice likely to be affected than females [9]. It has been reported, although quite rare to have multiple distinct nidi within a carpal bone [4], most common localization of an osteoid osteoma is in the lower extremities with more than half of the cases reported occurring in the femur and tibia.

Given that pain is the primary and often only symptom of osteoid osteoma, it is possible that it can be misdiagnosed as carpal tunnel syndrome, de Quervain's tenosynovitis, arthritis, neuroma, or avascular necrosis. In this case, the patient had been incorrectly diagnosed with CMC arthritis [2]. Long-standing pain and brief but noticeable response to NSAIDs and pain at night are all clinical clues that can help direct a provider to an accurate diagnosis [11].

In this case, the lesion was not found upon first read of the original radiography due to the subtle appearance. However, since clinical suspicion was high for a diagnosis of osteoid osteoma, further imaging was ordered and resulted in an accurate diagnosis.

Treatment of an osteoid osteoma of the trapezium involves surgical removal of the nidus [2, 4]. Recurrence is rare and usually due to incomplete excision of the nidus by curettage [9]. Trapeziectomy was not indicated in this case because the lesion was small and did not extend to the trapeziometacarpal joint. Bone graft can also be considered when removing larger lesions, or excising the lesion may cause instability of the trapezium.

If surgery is contraindicated or a different treatment is desired, treatment with percutaneous trephination or drill resection with or without subsequent injection of ethanol has been reported. Other alternatives include laser photocoagulation and radiofrequency ablation [9].

In conclusion, diagnosis of osteoid osteoma can present as a diagnostic challenge. A high index of suspicion must be maintained for the diagnosis of osteoid osteoma of the trapezium in patients with long-standing wrist pain, given the rarity of this occurrence.
